# The History and Capabilities of Herbal Simples

**Published:** 1891-01-10

**Authors:** W. T. Fernie


### The History and Capabilities of Herbal Simples.

By W. T. Fernie, M.D.

### XXVI.—CURRANTS.

Some thirty-five years ago, at the time of the Crimean war, a patriotic song in praise of the French flag, and with a refrain of "Hurrah for the Red, White, and Blue," was most popular in our streets. Just now, I would paraphrase this with respect to the Currants of our gardens, and would say emphatically, "Hurrah! for the red, white, and black!" And, as we shall see presently, whilst the regimental tricolor of France gave distinction on the battlefield, so the brilliant hues of English currants have been acquired for promotion and success in the keen contest of vegetable life. The original currants in times past were small grapes, grown in Greece, at Zante, near Corinth, and hence called Corinthians; then they became "corantes," and eventually currants—

"Now will the Corinths, now the Rasps supply,
Delicious draughts."

But, as a proverb of that time pithily said, *Non cuivis homini contingit adire Corinthum*—"it was not for everyone to visit fashionable Corinth"; and therefore the name of currants became transferred in the provinces of the Epirus to certain small fruit closely resembling the grapes of Zante, but rather of the gooseberry order. These were identical with the red, white, and black currants which we now use in England for making pies, puddings, jams, and jellies. The bushes which produce them grow wild in the northern parts of Great Britain, and belong to the Saxifrage tribe of plants. Shakespeare, in the "Winter's Tale," makes Autolycus, the shrewd picker-up of unconsidered trifles, talk of "buying for the sheep-shearing feast three pounds of sugar, five pounds of currants, and rice."

Whilst the fruit of each sort is still unripe, so that its seeds, if planted, would not germinate, the berries remain green and sour. But as the seeds gain maturity, the pulp of the currants becomes soft and sweet; likewise the skins become brightly conspicuous in colour, so as to allure birds, who shall swallow the berries, and shall scatter the seeds in their droppings. Zeuxis, a famous Sicilian painter four hundred years before Christ, depicted grapes and currants with such fidelity that birds came to try and devour them from his canvas. Nowadays we may well pardon the petty depredations of blackbirds and thrushes in our gardens, because of the important part which the ancestors of these feathered gourmets have played in thus specialising our choicest fruits.

Originally all the currants in every single bunch were united in one common berry, then these presently became divided and brightly coloured, so as to make a more attractive multiple display.

White currants are the most simple in kind, and the red are a step in advance. If equal parts of either fruit and of sugar are put over the fire, the liquid which separates spontaneously will make a most agreeable jelly, because of the pecten with which it is furnished chemically. Nitric acid will convert this pecten into oxalic acid, or salts of sorrel. The juice of red currants also contains wholesome malic and citric acids. When made into a jelly with sugar it is excellent in fevers, and acts as an antiputrescent, if taken at table with venison, or hare, or other "high" meats.

The red currant—*Ribes rubrum*—is called Wineberry in the northern counties, or Garnetberry, from its rich red colour and transparency. Its sweetened juice is a favourite drink in Paris, being preferred there to orgeat. This especially suits persons of sanguine temperament. Red and white currants are really trustworthy remedies in most forms of obstinate visceral obstruction.

Black currants grow wild in British woods, from mid-Scotland southwards. Throughout Sussex and Kent the black-currant shrub is called "Gazles," as corrupted from the French "Groseilles"—gooseberries.

This fruit is cooling, laxative, and anodyne. Its thickened juice, concocted with, or without sugar, formed the "rob" of old English times. The black currant is often named by our peasantry "squinancy," or "quinsyberry," because a jelly prepared therefrom has been long employed for sore throat and quinsy. The leafglands of its young leaves secrete from underneath a fragrant odorous fluid. Therefore, if newly gathered, and infused for a moment in hot water, and then dried, they make an excellent substitute for tea. Also, the fresh leaf, when applied to a gouty part, will assuage pain and inflammation. In Russia the leaves are employed to fabricate brandy made from common spirit. A medicinal wine is also brewed there from the fruit together with honey. In this country we use a decoction of the leaf, or of the bark, as a gargle; and in Siberia black currants grow as large as hazelnuts. They are often combined by our herbalists with the seeds of the wild carrot for stimulating the kidneys in passive dropsy. Bergius called the leaf of the black currant *mundans, pellens, et diuretica*. Both the black and red currants afford a pleasant home-made wine—*Ex eo optimum vinum fieri potest non deterius vinis vetioribus viteis*, wrote Haller in 1750. White currants yield the best wine, and this may be improved by keeping, even for twenty years.

Dr. Thornton says, "I have used old wine of white currants for calculous affections, and it has exceeded all expectation."

Black currant jelly should not be made with too much sugar, or its curative virtues will be impaired. A teaspoonful of this may be given three or four times in the day to a child with thrush. The generic term "ribes" applied to all fresh currants is of Arabian origin, and signifies acidity. Grocers' currants still come from the Morea, being small grapes dried in the sun and put in heaps to cake together. Then they are dug out with a crowbar, and trodden into casks for exportation.

Our national plum pudding, of Christmas celebrity, can no more be made without these currants than little Tom Tucker, who sang for his supper, could cut his white bread without any knife or could contemplate wedlock without any wife. Former cooks made a strange use of such fruit, according to a poet of the Middle Ages (King), who says:—

"They buttered currants on fat veal bestowed,
And rumps of beef with virgin honey stewed."

These kitchen currants are the subject of another whimsical nursery rhyme, as old as the hills, with a riddling play on the dissyllabic word currant:—

"Higgledy, piggledy, here we lie,
Pick'd, and pluck'd, and put in a pie;
My first is snapping, snarling, growling;
My second's industrious, romping, prowling."

